# Mucinous Cystadenoma Causing Abdominal Distension: A Case Report

**DOI:** 10.7759/cureus.3657

**Published:** 2018-11-30

**Authors:** Alexandra M Craen, David Lebowitz, Kendra Amico, Latha Ganti

**Affiliations:** 1 Emergency Medicine, Ocala Regional Medical Center / University of Central Florida College of Medicine, Orlando, USA; 2 Emergency Medicine, University of Central Florida College of Medicine, Orlando, USA

**Keywords:** mucinous cystadenoma, ovarian mass, adnexal mass, benign neoplasm, abdominal distension, gynecological surgery, emergency medicine, cystic mass, abdominal pain, gynecologic cancer

## Abstract

Mucinous cystadenomas are a common benign neoplasm of the ovaries that can grow much larger than other adnexal masses. We report a case of a 28-year-old female who presented with one month of increasing abdominal distension and upper abdominal pain; she was found to have a 30-centimeter (cm) adnexal mass. Pathology showed a benign mucinous cystadenoma with no evidence of malignancy. The authors discuss the initial evaluation and management of adnexal masses in general, as the variety of etiologies and severity can make this a difficult task. The characteristics of different adnexal masses and the indications for gynecologic consultation and surgery are also discussed.

## Introduction

Adnexal masses can occur at any age of life and there is a wide variety of pathology, both gynecologic and nongynecologic. Imaging, lab markers, and possible surgical intervention with a pathologic study are necessary to properly diagnose these masses. Age and reproductive status can also help narrow down likely causes [1]. The risk for ovarian cancer increases after menopause: 70% of patients are 55 years and older. Women of reproductive age are more likely to have benign masses [2]. Mucinous cystadenomas are a common benign neoplasm of the ovaries and can become much larger compared to the more common serous cystadenomas [3-4]. Surgical intervention of adnexal masses is warranted if concern for malignancy exists or if the patient is symptomatic [1,5].

## Case presentation

 A 28-year-old, previously healthy woman presented to the emergency department (ED) with two months of abdominal distension and one week of upper abdominal pain. The distension had initially abated after two weeks, but then gradually worsened until presentation. Her upper abdominal pain worsened with movement and improved with sitting upright. She denied any nausea, vomiting, constipation, diarrhea, urinary symptoms, vaginal bleeding or discharge, or other complaints. She denied any prior history of abdominal distension or liver disease. She reported regular menstruation, and her last menstrual period was one week prior. Her past obstetric history was gravida three, para two, abortus one. She reported a family history of ovarian cancer and colon cancer in distant relatives.

**Figure 1:**
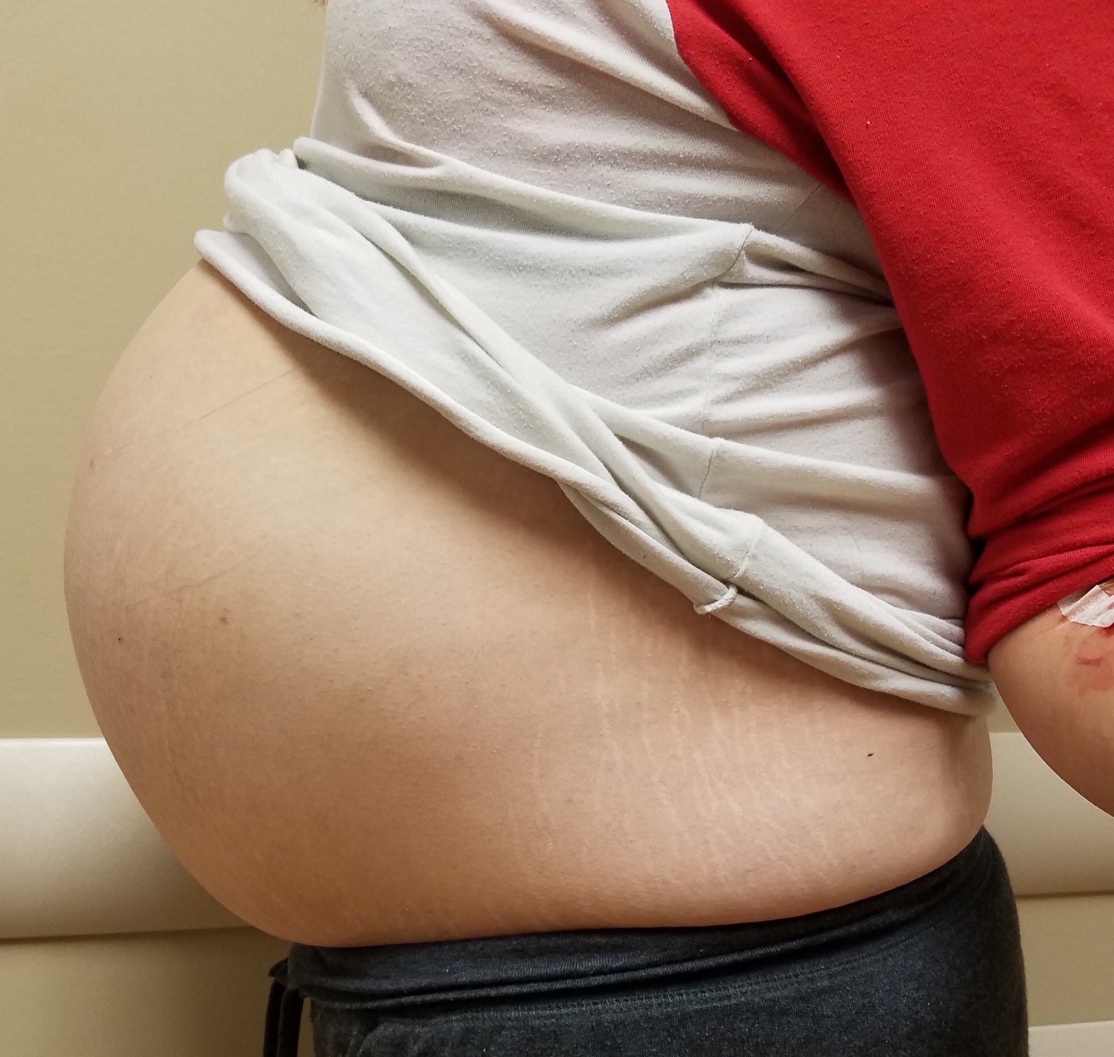
Abdominal distention of the patient.

Physical examination revealed a firmly distended abdomen with no fluid wave (Figure 1). There was no focal tenderness, rebound, or guarding of the abdomen. There were no skin changes or extremity edema noted. Cardiovascular and pulmonary exams were unremarkable. 

**Figure 2:**
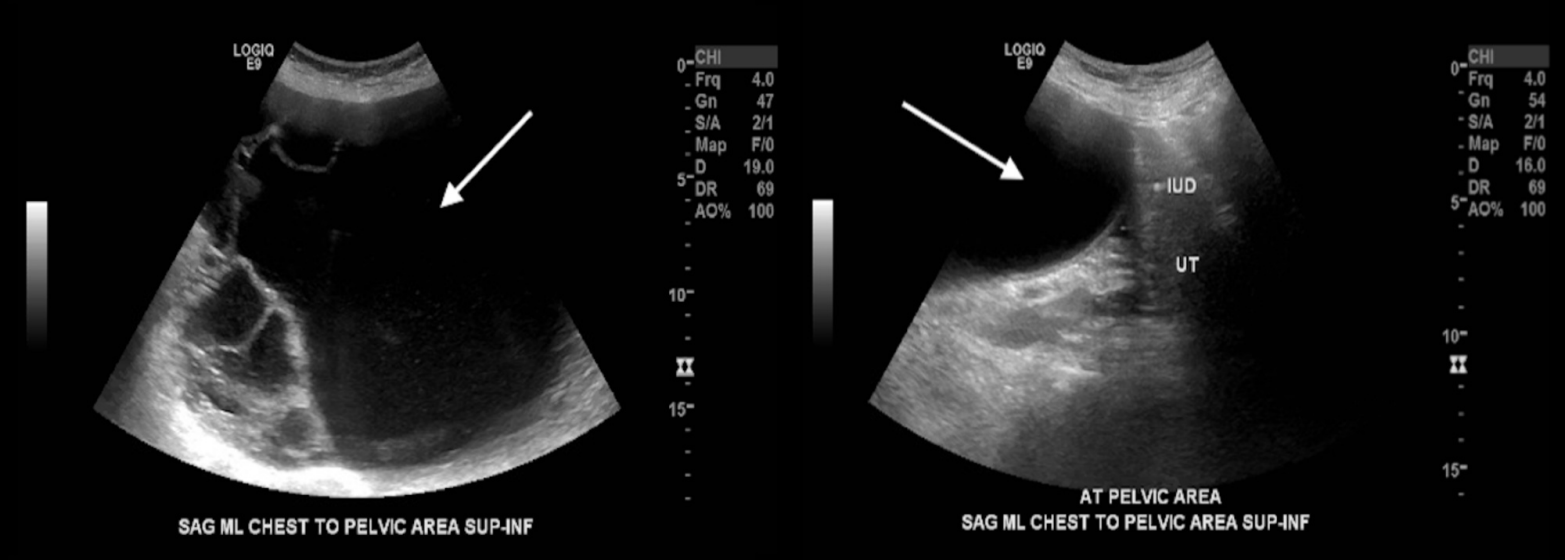
Transabdominal ultrasonography with two sagittal views of the upper abdomen and pelvic area showing a large cystic mass.

**Figure 3:**
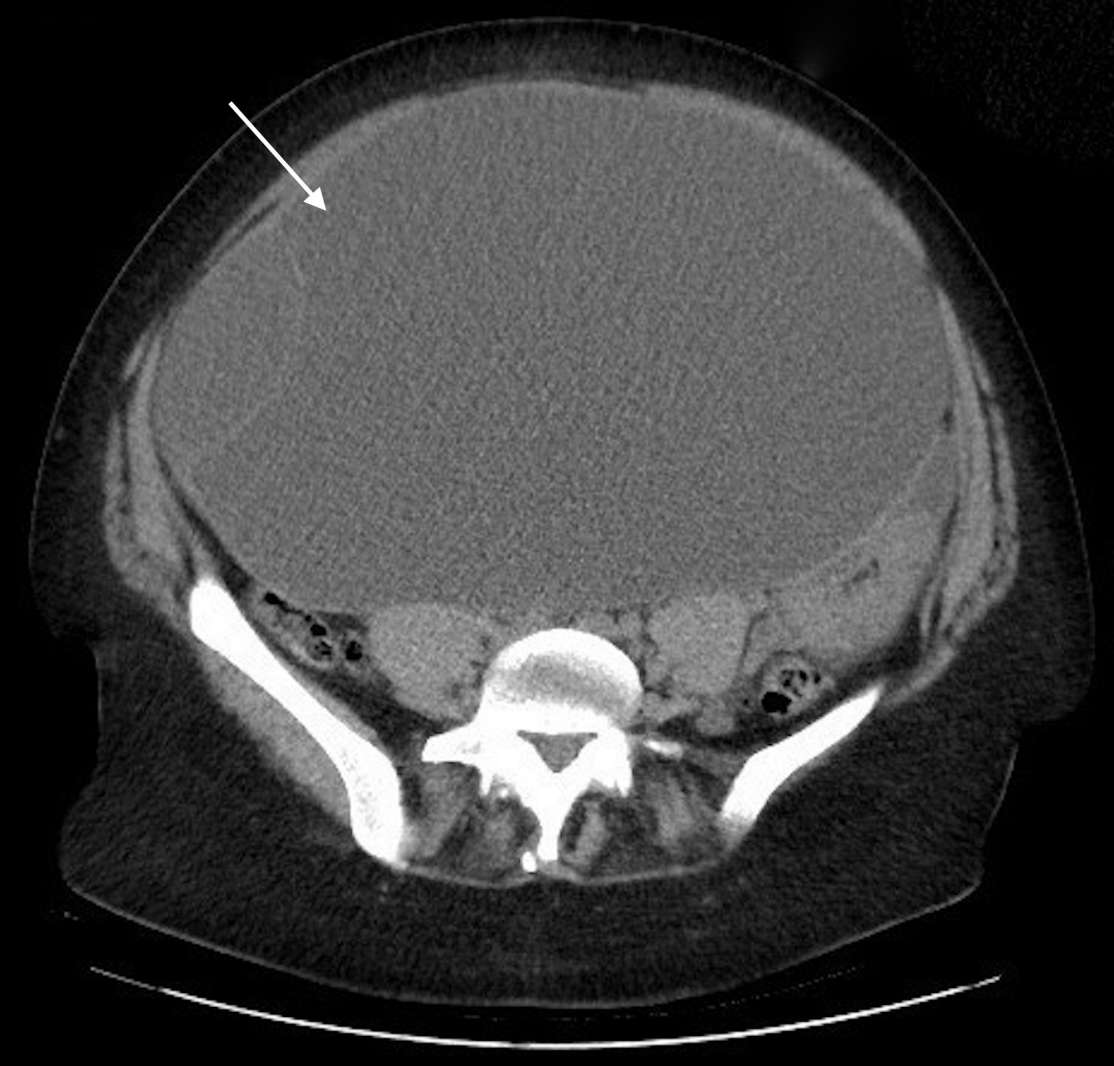
Axial computed tomography image of the abdomen at the level of the iliac crest showing a large cystic mass measuring up to 30 cm.

A point of care transabdominal ultrasound at the bedside showed several, large cystic structures and no obvious pregnancy. Laboratory studies were unremarkable and her beta-hcg returned negative. A comprehensive abdominal ultrasound showed a large cystic mass arising from the chest to the pelvic area (Figure 2). As the source of the mass was unclear, a computed tomography (CT) scan of the abdomen and pelvis was performed and showed a multi-septated cystic mass, measuring 30.0 x 28.9 x 19.0 cm, arising from one of the adnexal regions (Figure 3). Gynecology was consulted and performed a laparotomy with left salpingo-oophorectomy the following day. A 30 cm adnexal mass was removed. Surgical pathology revealed a mucinous cystadenoma with no cytologic malignancy found. The patient made a full recovery.

## Discussion

Adnexal masses can be difficult to evaluate and manage because of the large differential diagnosis and variable urgency associated with differing etiologies. In the emergency department, a relevant history should also include risk factors for cancer. A family history of ovarian or breast cancer is a strong risk factor for a patient developing ovarian cancer with an increase from 1.6% to about 5% with one relative [6]. Other oncologic risk factors include nulliparity, infertility, early menstruation or late menopause, white race, and endometriosis [1,6]. A full physical examination is also warranted in any patient with abdominal pain or concern for malignancy. While the pelvic examination has not been shown to identify adnexal masses well, it is still recommended to assess for other etiologies [7]. It is important to consider emergent diagnoses, such as ectopic pregnancy, tubo-ovarian abscess, and adnexal mass with associated ovarian torsion [1].

Transvaginal ultrasonography is the recommended and most commonly used imaging modality to evaluate an adnexal mass [1]. Malignant characteristics on ultrasound include a size greater than 10 cm, irregularity, papillary or solid structures, ascites, and high color Doppler flow on ultrasound. Benign characteristics include simple, thin-walled structures with no solid components or internal blood flow on Doppler ultrasound [8-9]. Masses believed to be benign, which are asymptomatic, may be observed with repeated transvaginal ultrasound rather than surgery [1,5].

A CT scan was done in our ED after ultrasonography to better assess the source and size of the mass. CT scans can be helpful in detecting lymph node enlargement, metastases, ascites, and possible alternative primary sites of tumors [10]. The CT returned, showing a 30 cm multi-septated cystic mass arising from one of the adnexa. Some experts consider cysts greater than 10 cm an indication for surgery [1,11]. Our patient’s mass was more than double this, prompting the gynecology team to take the patient to surgery the next day.

The pathology results showed a mucinous cystadenoma, which is a common benign adnexal neoplasm. They are larger and more likely to be multiloculated compared to serous cystadenomas, the most common benign neoplasm [4]. Other common benign adnexal masses in adult females include dermoid cysts and Brenner tumors [3]. Although benign, symptomatic patients experiencing pelvic pain, urinary symptoms, or associated ovarian torsion may have them surgically removed.

## Conclusions

Adnexal masses have a large variety of etiologies that can be difficult to diagnose on initial presentation. Mucinous cystadenomas are a type of benign adnexal neoplasm that can grow much larger than other masses. This caused the abdominal distension and pain seen in the patient discussed. The management of benign masses depends on the concern for malignancy, size, and patient’s symptoms. Point of care or bedside ultrasound can guide in medical decision-making or diagnostics, many times enhancing our process by helping us decide which is the best next test or step in care.
